# The *Drosophila* Mutagen-Sensitivity Gene *mus109* Encodes *DmDNA2*

**DOI:** 10.3390/genes13020312

**Published:** 2022-02-07

**Authors:** Chandani Mitchell, Vada Becker, Jordan DeLoach, Erica Nestore, Elyse Bolterstein, Kathryn P. Kohl

**Affiliations:** 1 Biology Department, Winthrop University, Rock Hill, SC 29733, USA; mitchellc27@winthrop.edu (C.M.); deloachj4@winthrop.edu (J.D.); nestoree2@winthrop.edu (E.N.); 2 Biology Department, Northeastern Illinois University, Chicago, IL 60625, USA; vabecker@uw.edu (V.B.); e-bolterstein@neiu.edu (E.B.)

**Keywords:** *mus109*, DNA2, DNA repair

## Abstract

The identification of mutants through forward genetic screens is the backbone of *Drosophila* genetics research, yet many mutants identified through these screens have yet to be mapped to the *Drosophila* genome. This is especially true of mutants that have been identified as mutagen-sensitive (*mus*), but have not yet been mapped to their associated molecular locus. Our study addressed the need for additional *mus* gene identification by determining the locus and exploring the function of the *X*-linked mutagen-sensitive gene *mus109* using three available mutant alleles: *mus109^D1^*, *mus109^D2^*, and *mus109^lS^*. After first confirming that all three *mus109* alleles were sensitive to methyl methanesulfonate (MMS) using complementation analysis, we used deletion mapping to narrow the candidate genes for *mus109.* Through DNA sequencing, we were able to determine that *mus109* is the uncharacterized gene *CG2990,* which encodes the *Drosophila* ortholog of the highly conserved DNA2 protein that is important for DNA replication and repair. We further used the sequence and structure of DNA2 to predict the impact of the *mus109* allele mutations on the final gene product. Together, these results provide a tool for researchers to further investigate the role of DNA2 in DNA repair processes in *Drosophila.*

## 1. Introduction

The development of gene mapping techniques has a long and storied history in the *Drosophila melanogaster* model system (reviewed in [1]), beginning with Alfred Sturtevant’s fundamental publication of the first genetic map in 1913 [2]. In this work, Sturtevant showed that genes are arranged in a linear order along chromosomes and that the recombination frequency between two genes could be used as a measure of the distance between them. This discovery created the foundation for other key advances in *Drosophila* gene mapping, including the generation of detailed polytene chromosome cytogenetic maps [3,4], the development of deletion kits covering the genome [5,6,7], and the sequencing of the *D. melanogaster* genome [8].

However, despite these advances, the current *D. melanogaster* genome annotation includes 14,184 genes that have not yet been mapped to the molecular genome (FlyBase R6.43; [9]), including many genes that were discovered in forward genetic screens. In these cases, alleles have been discovered that produce a phenotype of interest, but the molecular locus responsible for this phenotype remains unknown. For example, several forward genetic screens have been conducted to identify *D. melanogaster* mutants with defects in DNA repair (e.g., [10,11,12,13,14,15]). In these screens, flies that showed reduced survival in the presence of a mutagen—usually the alkylating agent methyl methanesulfonate (MMS)—were identified as probable DNA repair mutants. To date, 58 of these mutagen-sensitive (*mus*) stocks have been generated, yet the gene responsible for the *mus* phenotype is known for only 15 of these stocks [16,17]. Importantly, each mapped *mus* gene has encoded an ortholog of a human DNA repair protein [17], including proteins implicated in disorders such as Bloom syndrome [18], Fanconi anemia [16], and xeroderma pigmentosum [19].

The knowledge derived from studies of these 15 mapped *mus* genes demonstrates the utility of mapping *mus* genes to facilitate DNA repair research in *Drosophila*. With this in mind, we sought to map *mus109*, an *X*-linked essential gene with three extant alleles: *mus109^D1^* [13] and *mus109^D2^* [14] are homozygous viable hypomorphic alleles, whereas *mus109^lS^* is a homozygous lethal null allele [20]. *mus109* mutants are characterized by chromosomal instability in the absence of mutagen treatment [20,21,22,23], with the majority of chromosome breaks occurring in heterochromatin [22]. Further, *mus109* mutants are sensitive to MMS, 4-nitroquinoline-1-oxide (4NQO), and γ irradiation [13,14,24,25], which are mutagens that create DNA adducts (MMS and 4NQO; [26,27]) and oxidative damage (γ irradiation; [28]). In this manuscript, we present detailed mapping data obtained through complementation analysis, deletion crosses, and DNA sequence alignment showing that *mus109* is the uncharacterized *Drosophila* gene *CG2990* (human *DNA2*). We further discuss the potential functionality of the *mus109* mutant alleles by comparing the mutations to conserved catalytic regions in DNA2.

## 2. Materials and Methods

### 2.1. Drosophila Stocks and Maintenance

*D. melanogaster* stocks were maintained at 25 °C in bottles containing Nutri-Fly Bloomington Formulation media (Genesee Scientific) with a 12h day/night cycle. Experimental crosses were conducted in narrow vials containing corn syrup/soy media (Archon Scientific). The following fly stocks were obtained from the Bloomington Drosophila Stock Center (BDSC): *mus109^D1^* (BDSC# 2320), *mus109^D2^* (BDSC# 2307)*, mus109^lS^* (BDSC# 4168), *Df(1)ED6991* (BDSC# 37535), *Df(1)ED6989* (BDSC# 9056), *Df(1)BSC539* (BDSC# 25067), *Df(1)BSC754* (BDSC# 26852), *DGRP-59* (wild-type; BDSC# 28129), and *FM7c, P{GAL4-Kr.C}DC1, P{UAS-GFP.S65T}DC5, sn^+^* (BDSC# 5193).

### 2.2. Complementation Analysis

Five *mus109* heterozygous females—carrying either the *mus109^D1^*, *mus109^D2^*, or *mus109^lS^* chromosome over an *X* chromosome balancer marked with the dominant Bar eye phenotype—were crossed to five hemizygous *mus109^D1^* or *mus109^D2^* males per vial to establish Brood 1 (day 0). On day 3, the flies were flipped into new vials to establish Brood 2. On day 4, Brood 1 vials were mock treated with 250 µL water. On day 5, the adult flies were discarded from Brood 2 vials, and on day 6, Brood 2 vials were treated with 250 µL 0.05% methyl methanesulfonate (MMS; Sigma-Aldrich). Adult offspring were frozen on day 18 (Brood 1) or day 21 (Brood 2) and were subsequently scored for sex and eye phenotype. For each vial, relative survival was calculated as the ratio of *mus109* mutant to non-mutant flies in Brood 2, normalized to the same ratio in the corresponding Brood 1 vial. Vials with fewer than 15 progeny in either Brood 1 or 2 were excluded from analysis, as in [29]. Statistical significance was determined by one-way ANOVA with Tukey’s correction for multiple comparisons. Statistical analysis and graphing were performed using GraphPad Prism 7.05.

### 2.3. Deletion Mapping

Four deletions covering the area predicted by Mason et al. [14] to contain *mus109* were selected: *Df(1)ED6991*, *Df(1)ED6989*, *Df(1)BSC539*, and *Df(1)BSC754*. Five heterozygous females—carrying one of the deletions over an *X* chromosome balancer—were crossed to five *mus109^D2^* males per vial to establish Brood 1 (day 0). The remainder of the MMS sensitivity assay proceeded as in the complementation analysis crosses.

### 2.4. DNA Sequencing

For the *mus109^D1^* and *mus109^D2^* alleles, DNA was extracted from single adult hemizygous males using the protocol described in [30]. For *mus109^lS^*, flies were balanced with the *FM7c, P{GAL4-Kr.C}DC1, P{UAS-GFP.S65T}DC5, sn^+^* chromosome and homozygous third-instar larvae were identified by the absence of green fluorescence. DNA was then extracted from single homozygous third-instar larvae using the same protocol described in [30]. From these extracts, the *CG2990* coding region was amplified, purified with a GeneJet Gel Extraction Kit (Thermo Scientific), and sequenced (Eurofins Genomics). The primers used in PCR and sequencing are shown in Table S1. Sequences were aligned to the FlyBase [31] *CG2990* reference sequence and identified mutations were confirmed on a second DNA sample.

### 2.5. Protein Alignment

Clustal Omega [32] was used to conduct amino acid sequence alignment between DNA2 orthologs in wild-type *D. melanogaster* (NCBI Accession# NP_727386), *Mus musculus* (NCBI Accession# NP_796346.2)*, Homo sapiens* (NCBI Accession# NP_001073918.2), and *Caenorhabditis elegans* (NCBI Accession# NP_496516.1). Alignment was visualized using Jalview version 2.11.1.4 [33]. Domains were mapped and analyzed according to the domain map devised by Zhou et al. [34] based on the structure of *M. musculus* DNA2.

## 3. Results and Discussion

**Figure 1:**
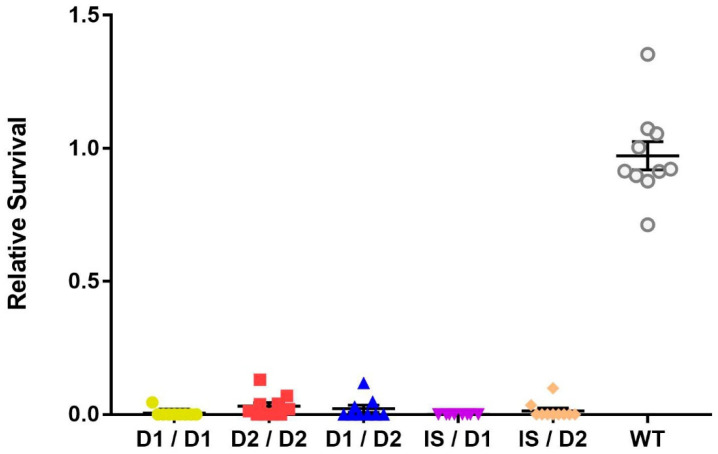
Relative survival of flies exposed to 0.05% methyl methanesulfonate for the indicated *mus109* allelic combinations and wild-type (WT). *mus109^lS^/mus109^lS^* could not be tested because the *mus109^lS^* allele is homozygous lethal. Each point represents one vial containing between 16 and 134 progeny (average = 55 progeny across all Brood 2 vials of all genotypes). The large horizontal line is the mean, while the upper and lower lines show the standard deviation.

Since the three *mus109* alleles—*mus109^D1^*, *mus109^D2^*, and *mus109^lS^*—were identified in the early 1980s [14,20,24], we first used complementation analysis to confirm that the fly stocks were still mutagen-sensitive. All possible *mus109* allelic combinations showed sensitivity to MMS with significantly lower relative survival values compared to wild-type (one-way ANOVA, F(5,50) = 255.7, *p* < 0.0001; Figure 1). Although the relative survival values were not significantly different between the *mus109* allele combinations (*p* = 0.221), the relative survival values were lower in genotypes containing *mus109^lS^* than in combinations without *mus109^lS^* (Figure S1), consistent with previous suggestions that *mus109^lS^* is amorphic [20] whereas *mus109^D1^* and *mus109^D2^* are hypomorphic [21]. 

**Figure 2:**
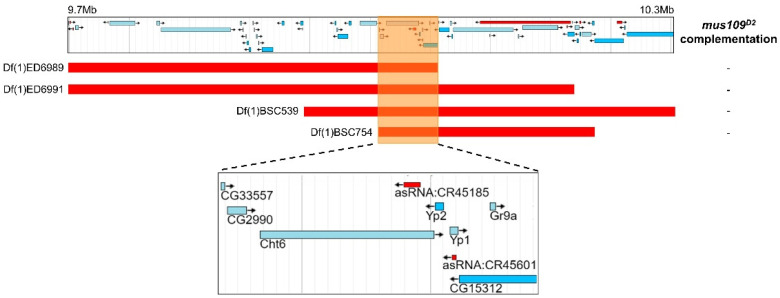
Results of deletion mapping assay where four deletions were crossed to *mus109^D2^.* Each deletion is shown as a red bar aligned with its genomic location on the *Drosophila melanogaster X* chromosome in the jBrowse [40] screenshot above. The orange box highlights the region of overlap between the four deletions, and the jBrowse area within this box is enlarged in the inset below. This insert shows the nine genes contained in the overlapping region. “-” indicates non-complementation of a deletion with *mus109^D2^.*

**Figure 3:**
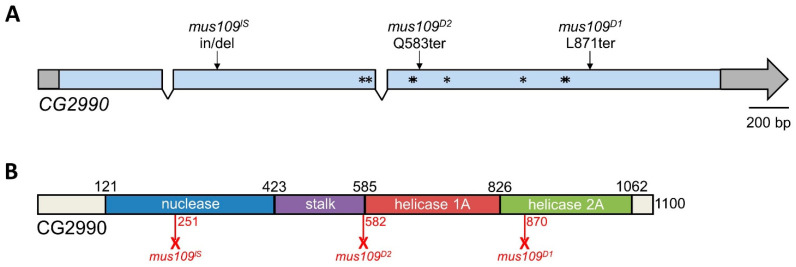
*CG2990* gene and protein structure. (**A**) *CG2990* contains three exons and two introns. Untranslated regions are shown in gray. Asterisks represent the missense mutations found in *mus109^D1^* mutants: E500D, L512F, S571G, E572Q, I633V, C755S, E825A, and K826E. *mus109^D1^* and *mus109^D2^* are truncated by nonsense mutations (L871ter and Q583ter, respectively), while *mus109^lS^* is truncated by a stop codon created by a 40 nucleotide deletion and four nucleotide (GAGG) insertion after amino acid 251. (**B**) CG2990 protein structure. The location of the nuclease, stalk, helicase 1A, and helicase 2A domains as identified in mouse DNA2 by Zhou et al. [34] are shown. Numbers above the bar denote the amino acid position of domain boundaries. Red numbers below the bar represent the predicted length of the CG2990 protein generated in each *mus109* mutant. Full-length CG2990 is 1100 amino acids long.

Next, deletion mapping was used to narrow the genomic location of *mus109*. Four deletions spanning the approximately 630 kb region predicted to contain *mus109* [14] were each crossed to *mus109^D2^* and assayed for sensitivity to MMS. With relative survival values of 0 in each case (Table S2), all four deletions failed to complement *mus109^D2^*. Thus, the location of *mus109* was narrowed to the approximately 62kb region shared by all deletions (Figure 2). The FlyBase entries for the nine genes within this region were reviewed to identify genes involved in DNA metabolism (Table 1), a characteristic of all mapped *mus* genes. Notably, one of these genes, *CG2990*, is orthologous to the well-characterized DNA repair gene *DNA2* [17]. Similar to *Drosophila mus109*, *DNA2* is essential in yeast and mice [35,36], its downregulation causes genome instability in yeast and human cells [37,38], and yeast *Dna2* mutants are sensitive to MMS [39]. Collectively, these observations suggested that *CG2990* is an ideal *mus109* candidate gene. To test our hypothesis that *mus109* was *CG2990*, we sequenced the *CG2990* coding region in wild-type flies and in each of the three *mus109* alleles. In comparing these sequences, we identified mutations resulting in premature stop codons in all three *mus109* alleles as well as eight missense mutations in *mus109^D1^* (Figure 3A), all of which likely affect the functionality of the *mus109* gene product.

The DNA2 protein is an essential and conserved nuclease-helicase with roles in several pathways that are crucial for maintaining genome integrity (reviewed in [41]). These pathways include long-track end resection during homologous recombination [42], Okazaki fragment processing [43], the recovery of stalled replication forks [44], and the maintenance of mitochondrial DNA [45]. Underscoring the importance of this protein, human DNA2 mutations have been implicated in mitochondrial myopathy [46], microcephalic primordial dwarfism [47], and some cancers [48]. DNA2 consists of a structure-specific nuclease and helicase/DNA-dependent ATPase connected by a β-barrel stalk [34]. While the nuclease activity is most critical to DNA2 repair functions [49,50,51], the helicase domains contribute to the narrow tunnel-like structure of DNA2 that allows single-stranded DNA access to the nuclease [34].

To explore the possible impact of the nonsense and missense mutations on *mus109* mutant allele functionality, we mapped DNA2 domains onto CG2990 using the mouse DNA2 protein structure [34] (Figure 3B). Like mouse DNA2, CG2990 contains a structure-specific nuclease domain and a helicase/DNA-dependent ATPase domain connected by a β-barrel stalk sequence, as well as two helicase motifs (1A and 2A) [34]. The two helicase motifs are common to members of the Upf1 subfamily of helicases and contain an ATPase at their cleft [52]; however, helicase and ATPase activity are considered weak and non-essential to DNA2 nuclease function [34,49]. 

**Figure 4:**
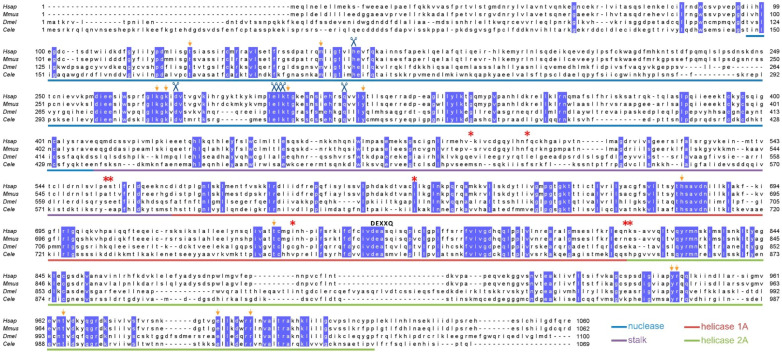
DNA2 amino acid sequence alignment. Residues conserved in all four species (*Homo sapiens (Hsap), Mus musculus (Mmus), D. melanogaster (Dmel),* and *Caenorhabditis elegans (Cele*)) are highlighted. Colored underlines represent the protein domains identified by Zhou et al. [34] in mouse DNA2. Conserved nuclease active sites are indicated by blue scissors, DNA contact site residues are indicated with orange arrows, and the DEXXQ-box helicase motif is indicated in bold text, all shown above the corresponding residues. Asterisks are located above the residues mutated in *mus109^D1^* mutants: E500D, L512F, S571G, E572Q, I633V, C755S, E825A, and K826E.

We further compared the CG2990 and human DNA2 protein sequences. The amino acid sequence alignment of CG2990 confirmed sequence homology to human DNA2 as well as with other model species (Figure 4). CG2990 contains the highly conserved DEXXQ-box helicase motif, as well as all known active site residues as defined in Zhou et al. [34]. Similarly, CG2990 contains most of the DNA contact site residues found in mouse DNA2 [34]. The insertion/deletion mutation in *mus109^lS^* creates a premature stop codon prior to the active site residues in the nuclease domain. This mutation likely abolishes nuclease function, which is known to be essential for viability in yeast [49]. If so, this could explain the homozygous lethal phenotype of *mus109^lS^* mutants. In contrast, the nonsense mutations in the *mus109^D1^* and *mus109^D2^* alleles occur after the conserved nuclease domain, which may allow for functional nuclease activity. While the nonsense mutation in *mus109^D1^* occurs in the second helicase domain, the I663V mutation in the stalk domain changes a highly conserved amino acid (Figure 4), which may impact protein folding and/or helicase functionality.

Considering our deletion mapping data and our identification of deleterious mutations in *CG2990*, we conclude that *mus109* is *CG2990*, the *Drosophila* ortholog of *DNA2* [17]. This knowledge will be immediately useful to the DNA repair community, as there are no existing non-transgenic alleles of *CG2990.* With the identification of three (two hypomorphic and one amorphic) alleles of *CG2990*, future genetic studies on the functions of DmDNA2 in DNA repair can be conducted. For example, comparisons between the *mus109^D1^* and *mus109^D2^* alleles exposed to mutagens that impact DNA replication could be used to dissect the function of the DmDNA2 helicase 1A domain, which is present in *mus109^D1^* but not *mus109^D2^*. Likewise, investigations of the *mus109^D1^* allele may further uncover the importance of the DmDNA2 helicase 2A domain, as this domain is not predicted to contribute to the tunnel structure needed for the nucleolytic activity of DNA2. Both of these genetic studies would also benefit from complementary biochemical analyses of the truncated DmDNA2 proteins produced in *mus109^D1^* and *mus109^D2^* mutants. Further, because DNA2 has been shown to act as a tumor suppressor (reviewed in [41]), the nuclease domain mutant allele may serve as a model with which to study DNA2-deficient cancer processes. Future studies may also aim to investigate genetic interactions with DmDNA2 by creating flies mutant in both *DNA2* and a critical gene in a redundant double-strand break repair pathway, such as *tosca* (*Exo1*). These and other experimental possibilities will greatly contribute to the growing body of work on DNA repair mechanisms and strengthen the use of *Drosophila* as a model for biomedical research.

## Figures and Tables

**Table 1:** Predicted function of genes within *mus109^D2^* and the non-complementing region.

Gene	Summary *
*asRNA:CR45185*	Antisense long non-coding RNA; function unknown.
*asRNA:CR45601*	Antisense long non-coding RNA; function unknown.
*CG2990*	5′-3′ DNA helicase, 5′-flap endonuclease; orthologous to HsDNA2 (DNA replication helicase/nuclease 2).
*CG15312*	Function unknown.
*CG33557*	DNA-binding transcription factor; orthologous to HsSCX (scleraxis bHLH transcription factor).
*Cht6*	Chitinase; orthologous to HsCHIT1 (chitinase 1).
*Gr9a*	Gustatory receptor.
*Yp1*	Yolk protein.
*Yp2*	Yolk protein.

* Information derived from gene ontology, summaries, and human orthologs sections of each gene’s FlyBase entry.

## Data Availability

The data presented in this study are available upon request from the corresponding author.

## Supplementary Materials

The following supporting information can be downloaded at: www.mdpi.com/article/10.3390/genes13020312/s1, Figure S1: Relative survival of flies exposed to 0.05% methyl methanesulfonate; Table S1: Primers used in this study. Table S2: Relative survival of deletions crossed to *mus109^D2^.*
