# Supplementary material for: The Drosophila Mutagen-Sensitivity Gene mus109 Encodes DmDNA2
Source: Genes (Basel). 2022 Feb 7;13(2):312. doi: 10.3390/genes13020312 (PMC8872385; doi:10.3390/genes13020312)
Supplement: Supplementary file 1 [file genes-13-00312-s001.zip › Genes Supplemental File Revision.pdf]

Supplementary Materials

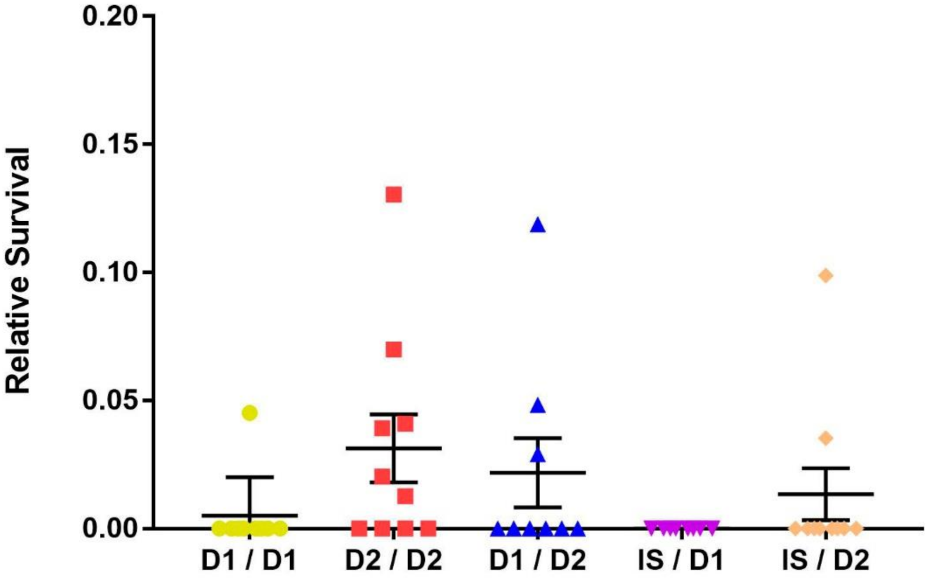

Figure S1. Relative survival of flies exposed to 0.05% methyl methanesulfonate for the indicated *mus109* allelic combinations. *mus109*^{IS}/*mus109*^{IS} could not be tested since the *mus109*^{IS} allele is homozygous lethal. Each point represents one vial containing between 16 and 78 progeny (average = 44 progeny across all Brood 2 vials of all genotypes). The large horizontal line is the mean, while the upper and lower lines show the standard deviation. Data are the same as in Figure 1, with wild-type excluded, and scale is modified to show values in detail.

Table S1. Primers used in this study.

Primer*	Sequence (5′ --> 3′)
DNA2 -87	CAGTCACTCTGTTCCCGCC
DNA2 1013	CCATGGAACACAAGGGCC
DNA2 1136a	CTCCCGCTAGCAACTTCTC
DNA2 1230a	CAGTGGATCCTCCTTGCTAGC
DNA2 1729	GTTCCACATCCCGTTTGGCCG
DNA2 1910	GAGGGAGAACGCTTCCAGGAG
DNA2 2081	GTTGATCAAGGGACTGCCCG
DNA2 2202a	GATTGTCCACAGCCGAATGCG
DNA2 2517	CCGCCGATTGTCAGATCC
DNA2 2576a	TTGGAATAAAGTCTCATCCG
DNA2 2667a	CATAGGTGAGCTCATTGGCC
DNA2 3160	CAGGTGGTGATTTCTCTGAC
DNA2 3516a	CATATCTCACTAAATCCC
DNA2 3677a	GAGGGGTCTTAAGTGAGG

* Primers are numbered according to their location with respect to the ATG of *CG2990*. Antisense primers are indicated with ‘a’.

Table S2. Relative survival of *X* chromosome deletions crossed to *mus109*^{D2}.

Deletion	Relative Survival	n*
Df(1)ED6989	0	978
Df(1)ED6991	0	1111
Df(1)BSC539	0	430
Df(1)BSC754	0	693

* n = total number of flies scored across all Brood 2 vials for that deletion.
