# Supplementary figures and images for: The Drosophila Mutagen-Sensitivity Gene mus109 Encodes DmDNA2
Source: Genes (Basel). 2022 Feb 7;13(2):312. doi: 10.3390/genes13020312 (PMC8872385; doi:10.3390/genes13020312)

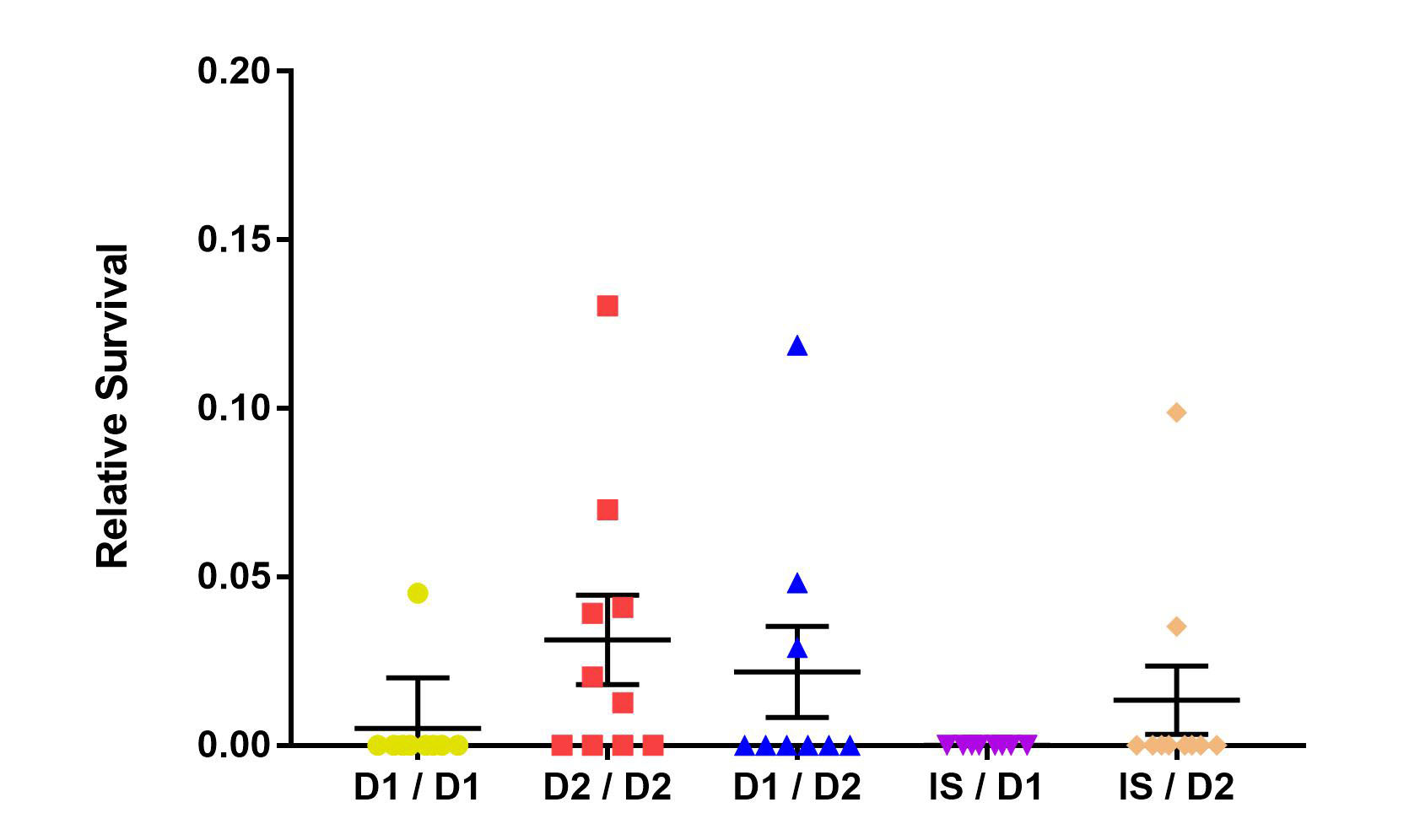

Supplement: Supplementary file 1 [file genes-13-00312-s001.zip › Figure S1.jpg]
